# Postprandial Regulation of Hepatic MicroRNAs Predicted to Target the Insulin Pathway in Rainbow Trout

**DOI:** 10.1371/journal.pone.0038604

**Published:** 2012-06-06

**Authors:** Jan A. Mennigen, Stéphane Panserat, Mélanie Larquier, Elisabeth Plagnes-Juan, Françoise Medale, Iban Seiliez, Sandrine Skiba-Cassy

**Affiliations:** UMR1067 Nutrition, Métabolisme, Aquaculture, Institut National de la Recherche Agronomique, Saint-Pée-sur-Nivelle, Pyrénées-Atlantiques, France; University of Texas Health Science Center at Houston, United States of America

## Abstract

Rainbow trout are carnivorous fish and poor metabolizers of carbohydrates, which established this species as a model organism to study the comparative physiology of insulin. Following the recent characterisation of key roles of several miRNAs in the insulin action on hepatic intermediary metabolism in mammalian models, we investigated the hypothesis that hepatic miRNA expression is postprandially regulated in the rainbow trout and temporally coordinated in the context of insulin-mediated regulation of metabolic gene expression in the liver. To address this hypothesis, we used a time-course experiment in which rainbow trout were fed a commercial diet after short-term fasting. We investigated hepatic miRNA expression, activation of the insulin pathway, and insulin regulated metabolic target genes at several time points. Several miRNAs which negatively regulate hepatic insulin signaling in mammalian model organisms were transiently increased 4 h after the meal, consistent with a potential role in acute postprandial negative feed-back regulation of the insulin pathway and attenuation of gluconeogenic gene expression. We equally observed a transient increase in *omy- miRNA-33* and *omy-miRNA-122b* 4 h after feeding, whose homologues have potent lipogenic roles in the liver of mammalian model systems. A concurrent increase in the activity of the hepatic insulin signaling pathway and the expression of lipogenic genes (*srebp1c*, *fas*, *acly)* was equally observed, while lipolytic gene expression (*cpt1a* and *cpt1b)* decreased significantly 4 h after the meal. This suggests lipogenic roles of *omy-miRNA-33* and *omy-miRNA-122b* may be conserved between rainbow trout and mammals and that these miRNAs may furthermore contribute to acute postprandial regulation of *de novo* hepatic lipid synthesis in rainbow trout. These findings provide a framework for future research of miRNA regulation of hepatic metabolism in trout and will help to further elucidate the metabolic phenotype of rainbow trout.

## Introduction

Maintenance of energy homeostasis in intermediary metabolism in vertebrates is critically regulated by insulin, which exerts actions on glucose and lipid metabolism in the liver [1]. Generally, insulin exerts anabolic effects with respect to these pathways, with a stimulatory effect on glycolysis and fatty acid synthesis, and a concurrent inhibition of gluconeogenesis and fatty acid oxidation [1]. Insulin exerts further anabolic effects at the level of protein metabolism, by stimulating protein synthesis and inhibiting autophagy [1], [2]. In pathological states, such as type-II diabetes, insulin signaling is impaired in liver cells, resulting in aberrant regulation of glucose and lipid metabolism [3]. This is characterized by an absence of inhibition on gluconeogenesis and fatty acid oxidation, resulting in development of hyperglycemia and hypertriglycemia [4].

**Figure 1:**
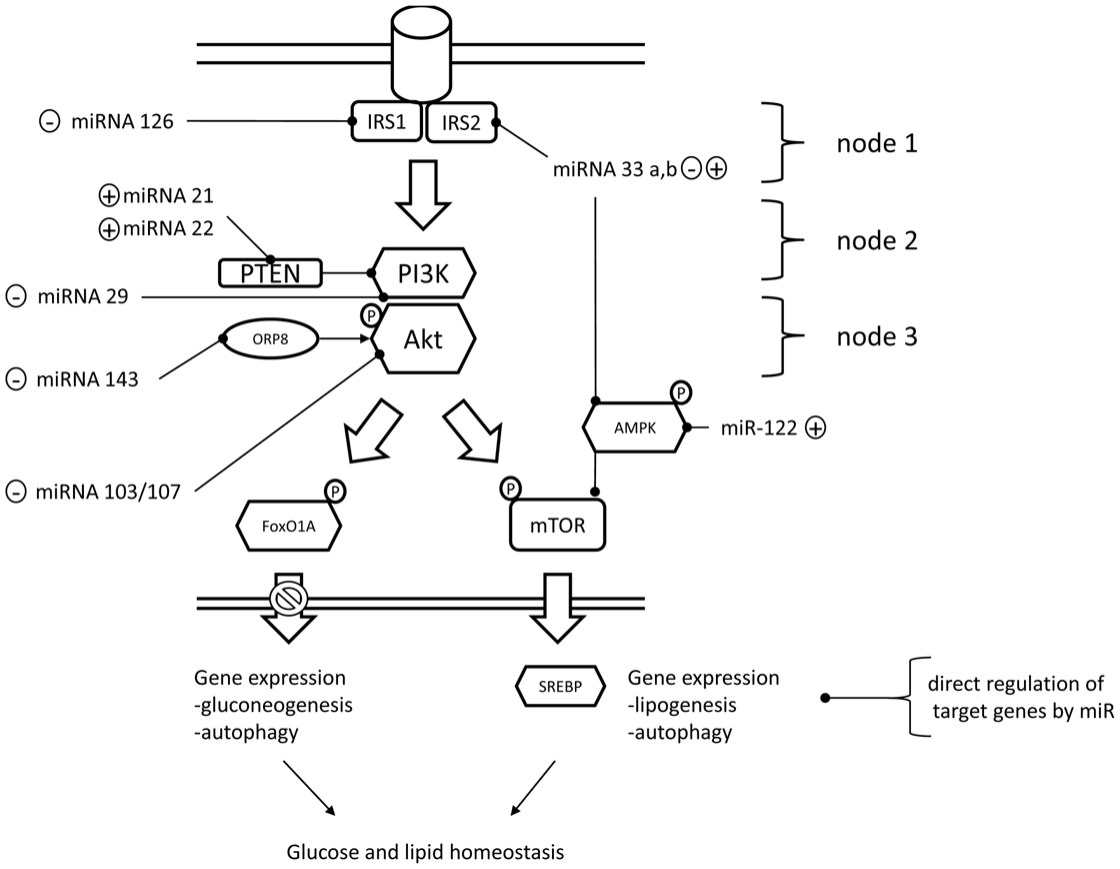
Schematic representation of experimentally validated actions of mammalian miRNAs on the mammalian hepatic insulin signaling pathway. Encircled + or – symbols indicate a stimulatory and inhibitory role on the insulin pathway in mammalian model systems. See text for explanations.

In recent mammalian literature [5], [6], [7], an involvement of miRNAs in the aetiology of insulin resistance in type-II diabetes has been hypothesized through the identification of altered hepatic expression of miRNAs in animal models of obesity and insulin-resistance. miRNAs are a family of short transcribed non-coding nucleotide sequences (∼21 nt), which are exported from the nucleus to bind to the mRNAs of target genes by complementary base-pairing mediated by the seed region of the miRNA [8]. The binding between miRNA and its specific mRNA target(s) results in the formation of a RISC (RNA-induced silencing complex), which, depending on the amount of complementarity of base pairing, causes degradation of the target mRNA or inhibition of its translation, respectively [8]. The identification of particular miRNAs as potential regulators of hepatic metabolism in mammals was followed by the demonstration of direct physiological functions of these individual mammalian miRNAs on components of the hepatic insulin pathway, which can be divided into several nodes [9], as depicted in **Fig. 1**. Briefly, insulin acts to recruit IRS proteins (node 1), which in turn recruit PI3K (node 2) to phosphorylate Akt (node 3). Downstream of Akt, the insulin pathway is bifurcated [10], and subsequent metabolic effects are to a large extent mediated by mTOR and FoxO1. The mTOR pathway subsequently regulates S6K1 and 4-EBP1 to stimulate hepatic protein synthesis and, at least partially, is involved in the stimulation of lipogenesis by stimulating SREBP1C-dependent gene expression [10], [11]. The FoxO1 pathway is primarily known to mediate insulin dependent repression of hepatic gluconeogenesis, at the level of gene expression [12]. Both, phosphorylation of mTOR and FoxO1 have been shown to inhibit hepatic autophagy [13], [14], and thereby further contribute to systemic amino acid, glucose [15], and lipid [16] homeostasis in mammals.

Several miRNAs can modulate protein abundance and/or phosphorylation status of key signaling components of the hepatic insulin pathway by acting at the level of different nodes. At the level of node 1 for example, *hsa*-*miRNA-126, hsa*-*miRNA-33a*, *hsa*-*miRNA-33b* act by inhibiting hepatic IRS1 and IRS2 protein abundance *in vivo* and *in vitro* [17], [18], [19]. At the level of node 3, *mmu*-*hsa-miRNA-29*, *mmu*-*miRNA-103, mmu*-*miRNA-107, mmu*-*miRNA-143* have been shown to inhibit Akt phosphorylation status *in vivo* and *in vitro* [20], [21], [22]. Conversely, *hsa-miRNA-21* and *hsa-miRNA-22* are known to target PTEN, a negative regulator of Akt phosphorylation status [23], [24] and over-expression of *hsa-miRNA-21* and *hsa-miRNA-22* has consequently been linked to increased insulin-stimulated Akt phosphorylation in the liver *in vitro* [23], [24]. Downstream of the third node, liver specific *mmu-miRNA-122* [25], as well as *mmu-miRNA-33* [18], [26] have been shown to be inversely correlated with the gene expression and/or phosphorylation status of the α-subunit of the metabolic sensor AMPK. Overall, the functions of the described miRNAs (**Fig. 1**) are consistent with postulated roles of miRNAs as feedback regulators, implicated in ‘fine-tuning’ regulation of target pathways [27]. Indeed, the insulin pathway in mammals has been predicted as a target pathway for miRNA mediated feedback [28]. In addition to regulating components of the insulin pathways, miRNAs can also directly act on downstream genes important in metabolism (**Table 1**), resulting in a complex regulatory network.

**Table 1:** Predicted target genes of fish homologues of human miRNAs known to act on the hepatic insulin pathway.

Target gene	*hsa-miRNA*	*gac-miRNA*	*ola-miRNA*	*dre-miRNA*	*tru-miRNA*	*tni-miRNA*
*Insulin signalling*						
*irs1*	*126*	*none*	*none*	*22,29*	*none*	*none*
*irs2*	*none*	*29,103,107*	*103,107*	*none*	*none*	*21*
*pi3k class1* *(various subunits)*	*103,107,143*	*21,22,33,103,107,126*	*21,33,126*	*none*	*21,29,33,143*	*29,107,143*
*Pten*	*none*	*103,107*	*103,107,143*	*none*	*none*	*none*
*mTOR*	*none*	*none*	*none*	*none*	*none*	*none*
*s6k* *(various subunits)*	*22,29,103,107*	*22,29,33,122*	*22,29,103,107,122*	*21,143*	*21,22,29,33,122*	*29,33,122*
*S6*	*none*	*none*	*126*	*none*	*none*	*none*
*4-ebp1*	*none*	*none*	*none*	*none*	*none*	*none*
*foxO1*	*none*	*21*	*21*	*none*	*none*	*none*
***Glucose metabolism***						
*m-pepck*	*33*	*none*	*21,33*	*none*	*22*	*33*
*g6pase*	*21,122*	*33*	*33*	*none*	*143*	*143*
*fbpase2*	*22,33*	*none*	*103,107*	*none*	*107*	*none*
***Lipid metabolism***						
*srebp1*	*none*	*none*	*none*	*none*	*none*	*none*
*Fas*	*103,107*	*122*	*none*	*none*	*122*	*none*
*Acly*	*103,107*	*103,107*	*103,107*	*none*	*none*	*none*
*Gapdh*	***122***	***29,33***	***none***	***none***	***none***	***143***
*cpt1a*	***33***	***none***	***none***	***103,107***	***none***	***none***
*cpt1b*	***103,107***	***none***	***none***	***none***	***none***	***none***
***Autophagy***						
*lc3b*	*none*	*none*	*none*	*none*	*none*	*none*
*atg4b*	*none*	*29*	*none*	*none*	*none*	*29*

The *in silico* prediction algorithms of miRNA targets available for fish included Japanese medaka (*Oryzias latipes),* the stickleback (*Gasterosteus aculeatus),* the Fugu pufferfish (*Takifugu rubripes)*, the green-spotted pufferfish (*Tetraodon nigroviridis)* and zebrafish (*Danio rerio)* and were used in addition to human (*Homo sapiens*) predictions as an approximation for *omy-miRNA* targets. All predictions are derived from the microCosm database.

Physiologically, the described miRNA-mediated inhibitions of components of the insulin signaling pathway result in measurable metabolic effects in mammalian model systems. Based on these metabolic effects, the described miRNAs can be functionally divided into two groups, acting primarily on glucose metabolism (**Fig. 1**, left handside) and lipid metabolism (**Fig. 1**, right handside). With respect to glucose metabolism, for example, *hsa-miRNA-29, hsa-miRNA-126, mmu-microRNA-103/107* generally favour hepatic glucose liberation, by inhibiting hepatic glucose storage [17] or favouring hepatic gluconeogenesis, at least partially by counteracting insulin-mediated repression of gluconeogenic genes *g6pase*, *pepck* and *fbpase* in the liver [20], [21]. Hepatic lipid metabolism is functionally regulated by *mmu-miRNA*-33 and *mmu-miRNA-122 in vivo* and *in vitro*, which favour lipogenesis through stimulating hepatic gene expression of *srebp1*, *acly* and *fas* while concurrently inhibiting fatty acid oxidation at least partially through the inhibition of *cpt1a* gene expression [18], [19], [25], [29]. As miRNAs act to inhibit target gene abundance or translation, the positive correlation between the expression of these miRNAs and the lipogenic target genes was speculated to be caused by targeting AMPK, an upstream repressor normally repressing the lipogenic pathway at the level of mTOR under low energy conditions [18], [25].

Based on these findings, we hypothesized that the hepatic expression of corresponding *omy-miRNA* homologues are acutely regulated in the postprandial regulation of intermediary metabolism in the liver of rainbow trout (*Oncorhynchus mykiss*). Rainbow trout emerged as an important model organism in the investigation of insulin signaling [30], [31]. Its glucose intolerant phenotype [32] makes the trout an important model for pathologies such as type II diabetes, characterized by prominent glucose intolerance [3]. Furthermore, its importance as an aquaculture species [33] has led to detailed investigations of the insulin pathway in trout, in order to understand the effect of nutrient composition on the insulin pathway and its subsequent regulation of intermediary metabolism [38]. Generally, insulin action in the liver of rainbow trout appears to be largely similar to mammals, inhibiting catabolic reactions while stimulating anabolic processes. Insulin activates key components of the hepatic intracellular signaling *in vitro* and *in vivo* [34], [35] resulting in metabolic effects mediated at the level of hepatic gene expression similar to the situation in mammals. For example, insulin inhibits gluconeogenic gene expression, decreases gluconeogenic enzyme activity [36], [37] and stimulates lipogenesis by the acute stimulation of lipogenic gene expression *in vitro* and *in vivo* [31], [37], [38]. Therefore, the importance of rainbow trout as a research model for insulin action with its detailed characterization of hepatic insulin signaling and function, as well as the recent identification of *omy-miRNA*s [33], make this species suitable to investigate the postprandial regulation of hepatic omy-miRNA in relation to insulin signaling and target genes.

[LOSSEST]Specifically, we first chose conserved *omy-miRNAs* based on the function of their human homologues on the insulin pathway and approximated the conservation of predicted targets through genome-based predictions for miRNA homologues in several fish species (**Table 1**). We then refed short-term fasted rainbow trout and measured the postprandial hepatic expression profile of *omy-miRNAs* (**Table 2**), the phospohorylation status of key signaling components of the insulin pathway (Akt, mTOR, S6, 4-EBP1, FoxO1) and metabolic gene targets, including genes involved in glucose metabolism (*pepck*, *g6pase1*, *g6pase2*, *fbpase2*) lipid metabolism (*srebp1c*, *fas*, *acly*, *gapdh, cpt1a*, *cpt1b*), and autophagy (*atg4b*, *lc3b*). This experimental design allows for a temporal resolution of multiple levels of control regulating the hepatic intermediary metabolism in trout.

**Table 2:** Primers and conditions for miRNA expression assays.

*miRNA target*	*Primer 5′ 3′ (FW)*	T
*omy-miRNA-21a*	TAGCTTATCAGACTGGTGTTGGC	62°C
*omy-miRNA-22*	TGCCAGCTGAAGAACTGT	60°C
*omy-miRNA-29a*	GCACCATTTGAAATCCAGTGT	62°C
*omy-miRNA-33*	GTCATTGTAGTTGCATTGA	61°C
*omy-miRNA-103*	AGCATTGTACAGGGCTATCA	60°C
*omy-miRNA-107*	AGCATTGTACAGGGCTATGA	64°C
*omy-miRNA-122a*	TGGAGTGTGACAATGGTGTTTT	60°C
*omy-miRNA-122b*	TGGAGTGTGACAATGGTGTCT	60°C
*omy-miRNA-126a*	TCGTACCGTGAGTAATAATGC	61°C
*omy-miRNA-143*	TGAGATGAAGCACTGTAGCTC	61°C

## Materials and Methods

### Prediction of MiRNA Target Genes in Fish

The target miRNAs investigated in this study were chosen based on their described effects on the mammalian insulin pathway in the liver. However, miRNAs can equally target downstream regulated genes of the insulin pathway directly. In order to predict the potential for *omy-miRNA*s to regulate both, components of the hepatic insulin pathway, as well as metabolic genes in trout, the online database microcosm (http://www.ebi.ac.uk./enrightsrv/microcosm/htdocs/targets/v5/) was used. The *in silico* prediction algorithms of miRNA targets available for fish species do not include rainbow trout, but include predictions for the stickleback (*Gasterosteus aculeatus),* the Japanese medaka (*Oryzias latipes)*, the zebrafish (*Danio rerio)* and two puffer fish species (*Takifugu rubripes* and *Tetraodon nigroviridis)*. Therefore, predictions for miRNA targets in these fish species were used in addition to human (*Homo sapiens*) predictions to serve as an approximation for potential target conservation in rainbow trout (**Table 1**).

### Fish and Experimental Protocol

The experiments were carried out in accordance with the clear boundaries of EU legal frameworks, specifically those relating to the protection of animals used for scientific purposes (i.e. Directive 2010/63/EU), and under the French legislation governing the ethical treatment of animals (Decret no. 2001-464, May 29^th^, 2001). The investigators carrying out the experiment had “level 1” or “level 2” certification, bestowed by the Direction Départementale des Services Vétérinaires (French vetinary services) to carry out animal experiments (INRA 2002-36, April 14^th^, 2002). The experiment was conducted at INRA St.Pée-sur-Nivelle, certified for animal services under the permit number A64.495.1 by the French vetinary services, which is the competent authority. Prior to the experiment, fish had initially been reared in our own experimental facilities (INRA, Donzacq, France) at 18°C and fed a commercial diet (Skretting, France; crude protein: 49.8% dry matter, crude fat: 13.8% dry matter; gross energy: 22 kJ/g dry matter). Immediately prior to the experiment, fish were fasted for 48 h, in order to allow for basal metabolite plasma concentrations to be reached. In trout, these basal metabolite concentrations are typically reached more slowly compared to endothermic mammals due to slower intestinal transit and gastric emptying. Following the fast, fish were fed once at libitum with the commercial diet. Six trout were sampled for each time point, starting with unfed fish at 0 h, and following feeding at 2 h, 4 h, 8 h, 12 h, 16 h and 24 h. Immediately following complete anaesthesia, which was confirmed by a complete absence of breathing or swimming response, trout were killed by a sharp blow to the head and decapitated to ensure fish were dead. Gut content of the sampled animals was checked to verify that fish had effectively consumed the diet. Blood was taken from the caudal vein and centrifuged (3000 g, 5 min) and the plasma recovered was immediately stored at −20°C. The liver was dissected and frozen in liquid nitrogen prior to storage at −80°C.

### Relative Gene Expression Analysis of Hepatic MiRNA and MRNA

Relative hepatic gene expression was determined by quantitative real-time RT-PCR. The extraction of total RNA was performed using the Trizol reagent (Invitrogen, Carlsbad, CA, USA) according to the manufacturer’s instructions. An amount of 1 µg of total RNA was used for cDNA synthesis. The NCode™ VILO™ miRNA cDNA synthesis kit (Invitrogen) or the SuperScript III RNAseH- Reverse transcriptase kit (Invitrogen) with oligo dT primers (Promega, Charbonniéres, France) was used to synthesize cDNA (n = 6 for each time point) for miRNA and mRNA, respectively. For gene expression assays, forward primer sequences for miRNAs were taken directly from the sequence information provided by Salem and colleagues [33], or, if not available, designed based on miRNA sequences found in miRBase [39]. The reverse primer used for all miRNA expression analysis was provided by the manufacturer with the NCode™ VILO™ miRNA cDNA synthesis kit (Invitrogen). The primer sequences used in the real-time RT-PCR assays for miRNAs and metabolic genes are shown in **Table 2** and **Table 3**, respectively. For real-time RT-PCR assays of omy-miRNAs, the Roche Lightcycler 480 system was used (Roche Diagnostics, Neuilly sur Seine, France). The assays were performed using a reaction mix of 6 µl per sample, each of which contained 2 µl of diluted cDNA template, 0.12 µl of each primer (10 µM), 3.12 µl Light Cycler 480 SYBR® Green I Master mix and 0.76 µl DNAse/RNAse free water (5 Prime GmbH, Hamburg, Germany). The PCR protocol was initiated at 95°C for 10 min for initial denaturation of the cDNA and hot-start Taq-polymerase activation, followed by 45 cycles of a two-step amplification programme (15 s at 95°C; 40 s at 60–64°C), according to the primer set used (**Table 2**). Melting curves were systematically monitored (temperature gradient at 1.1°C/10 s from 65–94°C) at the end of the last amplification cycle to confirm the specificity of the amplification reaction. Each PCR assay included replicate samples (duplicate of reverse transcription and PCR amplification, respectively) and negative controls (reverse transcriptase- and cDNA template-free samples, respectively). The gene expression assays for the metabolic genes has been described previously [35] and were carried out using the icycler iQ™ real-time PCR detection system. Assays were performed using a reaction mix of 15 µl per sample, each containing 5 µl of diluted cDNA, 0.5 µl of each primer (10 µM), 7.5 µl of iQ™ SYBR® Green Supermix (BIORAD, Hercules, CA, USA) and 1.9 µl of DNAse/RNAse free water. The PCR protocol was initiated at 95°C for 3 min for initial denaturation of the cDNA and hot-start iTaq™ DNA polymerase activation and continued with 35 cycles of a two-step amplification programme (20 s at 95°C; 20 s at 56–60°C), according to the primer set used (**Table 3**). Melting curves were systematically monitored (temperature gradient at 0.5°C/10 s from 55–94°C) at the end of the last amplification cycle to confirm the specificity of the amplification reaction. Each PCR run included replicate samples and controls as described above. For the expression analysis of both, miRNA and mRNA, relative quantification of target gene expression was performed using the ΔCT method described by Pfaffl [40]. The relative gene expression of *ef1α* was used for the normalization of measured mRNAs, and miRNAs, respectively. The relative expression of *ef1α* did not significantly change over time (data not shown). The reference gene *ef1α* has been previously used as a reference gene for analysis of postprandial metabolic gene expression in trout *in vivo* [35]. In all cases, PCR efficiency (E) was measured by the slope of a standard curve using serial dilutions of cDNA. In all cases, PCR efficiency values ranged between 1.8 and 2.2.

**Table 3:** Primers and conditions for mRNA expression assays.

*mRNA target*	*Primer 5′3′ (FW)*	*Primer 5′3′ (RV)*	T
*Glucose Metabolism*			
*m-pepck*	GTTGGTGCTAAAAGGGGCACAC	CCCGTCTTCTGATAAGTCCAA	59°C
*g6pase1*	CTCAGTGGCGACAGAAAGG	TACACAGCAGCATCCAGAGC	55°C
*g6pase2*	CAGAAGAACGCCCACAGAGT	CAGAAGAACGCCCACAGACT	55°C
*fbpase2*	GCTGGACCCTTCCATCGG	CGACATAACGCCCACCATAGG	59°C
***Lipid Metabolism***			
*srebp1c*	GACAAGGTGGTCCCAGTTGCT	CACACGTTAGTCCGCATCAC	60°C
*Fas*	TGATCTGAAGGCCCGTGTCA	GGGTGACGTTGCCGTGGTAT	60°C
*acly1*	GCTTTTGCCACGGTGGTCTC	GCTTCCGCTACGCCAATGTC	59°C
*g6pdh*	CTCATGGTCCTCAGGTTTG	AGAGAGCATCTGGAGCAAGT	59°C
*cpt1a*	TCGATTTTCAAGGGTCTTCG	CACAACGATCAGCAAACTGG	55°C
*cpt1b*	CCCTAAGCAAAAAGGGTCTTCA	CATGATGTCACTCCCGACAC	59°C
***Autophagy-related genes***			
*lc3b*	GAACAGTTTGACCTGCGTGAA	TCTCTCAATGATGACCGGAATCT	57°C
*atg4b*	TATGCGCTTCCGAAAGTTGTC	CAGGATCGTTGGGGTTCTGC	58°C
***Reference genes***			
*eF1α*	TCCTCTTGGTCGTTTCGCTG	ACCCGAGGGACATCCTGTG	59°C

### Protein Extraction and Western Blotting

Frozen liver samples (∼300 mg) were homogenized on ice with an Ultraturrax homogenizer (IMLAB Sarl, Lille, France). During homogenization samples were kept in a buffer containing 150 mmol l^−1^ NaCl, 10 mmol l^−1^ Tris, 1 mmol l^−1^ EGTA, 1 mmol l^−1^ EDTA (pH 7.4), 100 mmol l^−1^ sodium fluoride, 4 mmol l^−1^ sodium pyrophosphate, 2 mmol l^−1^ sodium orthovanadate, 1% (v/v) Triton X-100, 0.5% (v/v) NP40-IGEPAL and a protease inhibitor cocktail (Roche, Basel, Switzerland). Homogenates were centrifuged at 1000 g for 30 min at 4°C and supernatants were then centrifuged for 45 min at 150.000 g. The resulting supernatants (n = 5 for each time point) were stored at −80°C. Protein concentrations were determined using the Bio-Rad Protein assay kit (BIO-RAD, Hercules, CA, USA). According to the protein, quantities of 5–20 µg protein per sample were subjected to SDS-PAGE and Western Blotting, using the appropriate antibodies. All primary antibodies used for analysis of the insulin signaling pathway were obtained from Cell Signaling technologies (Ozyme, Saint Quentin Yvelines, France) and have been shown to cross-react successfully with rainbow trout proteins of interest [34], [41], [42]. The specific dilutions used for the primary antibodies are shown in **Table 4**. All antibodies were raised in rabbit, and after final washing, membranes were incubated with an IRDye infrared secondary anti-rabbit antibody raised in goat (LI-COR Inc. Biotechnology, Lincoln, NE, USA). Bands were visualized and quantified by Infrared fluorescence using the Odyssey® Imaging System (LI-COR Inc. Biotechnology, Lincoln, NE, USA).

**Table 4:** Primary antibodies and specific dilutions used in Western Blot analysis.

Protein target	Primary AB source	Dilution of primary AB
*Insulin signaling*		
Akt-p	Cell Signaling 9272	1∶1000
Akt	Cell Signaling 9271	1∶1000
mTOR-p	Cell Signaling 2972	1∶1000
mTOR	Cell Signaling 2971S	1∶1000
S6-p	Cell signaling 4856S	1∶1000
S6	Cell Signaling 2217S	1∶1000
4EBPI-p	Cell Signaling 9452S	1∶1000
4EBP1	Cell Signaling 9451S	1∶1000
FoxO1-p	Cell Signaling 9464	1∶500
FoxO1	Cell Signaling 9454	1∶500

### Statistical Analysis

Data were analyzed by univariate ANOVA. In cases where data were nonparametric or not homoscedastic, data transformations were used to meet ANOVA criteria. Normality was assessed using the Shaprio-Wilk test, while equality of variance was determined using Levene’s test. Following univariate ANOVA analysis, the Student-Newman-Keuls test was used for post-hoc analysis. Data was analysed using the SPSS software version 17.0.

## Results

### Predicted Conservation of Predicted Fish MiRNA Targets Compared to Humans

With respect to the insulin pathway, several miRNAs appear to have conserved predicted targets between fish and humans (**Table 1**). This is especially evident with regard to the targeting of PI3K kinase, which across the different fish species is predicted to be targeted by all miRNAs predicted in humans, including homologues of miRNA-103, miRNA-107 and miRNA-143. Additionally, in fish, miRNA-21, miRNA-22, miRNA-29 and miRNA-126 are predicted to target components of the insulin pathway upstream of node 3. However, differences in the predictions between humans and fish and between fish species exist, which is also evident for other components of the insulin signaling pathway downstream of the third node. In lipid metabolism related genes, *acly* is predicted to be targeted directly by miRNA-103/107 homologues in both humans and stickleback and medaka fish species. Of the metabolic genes involved in glucose metabolism, *m-pepck* is predicted to be targeted by miRNA-33 homologues in both humans and medaka and fugu fish species. Of the genes involved in autophagy, neither *lc3b* nor *atg4* are predicted to be targeted by the investigated miRNA in humans, but miRNA-29 homologues are predicted to target *lc3b* in two fish species, stickleback and fugu.

### Postprandial Regulation of MicroRNAs Involved in Hepatic Metabolism

**Figure 2:**
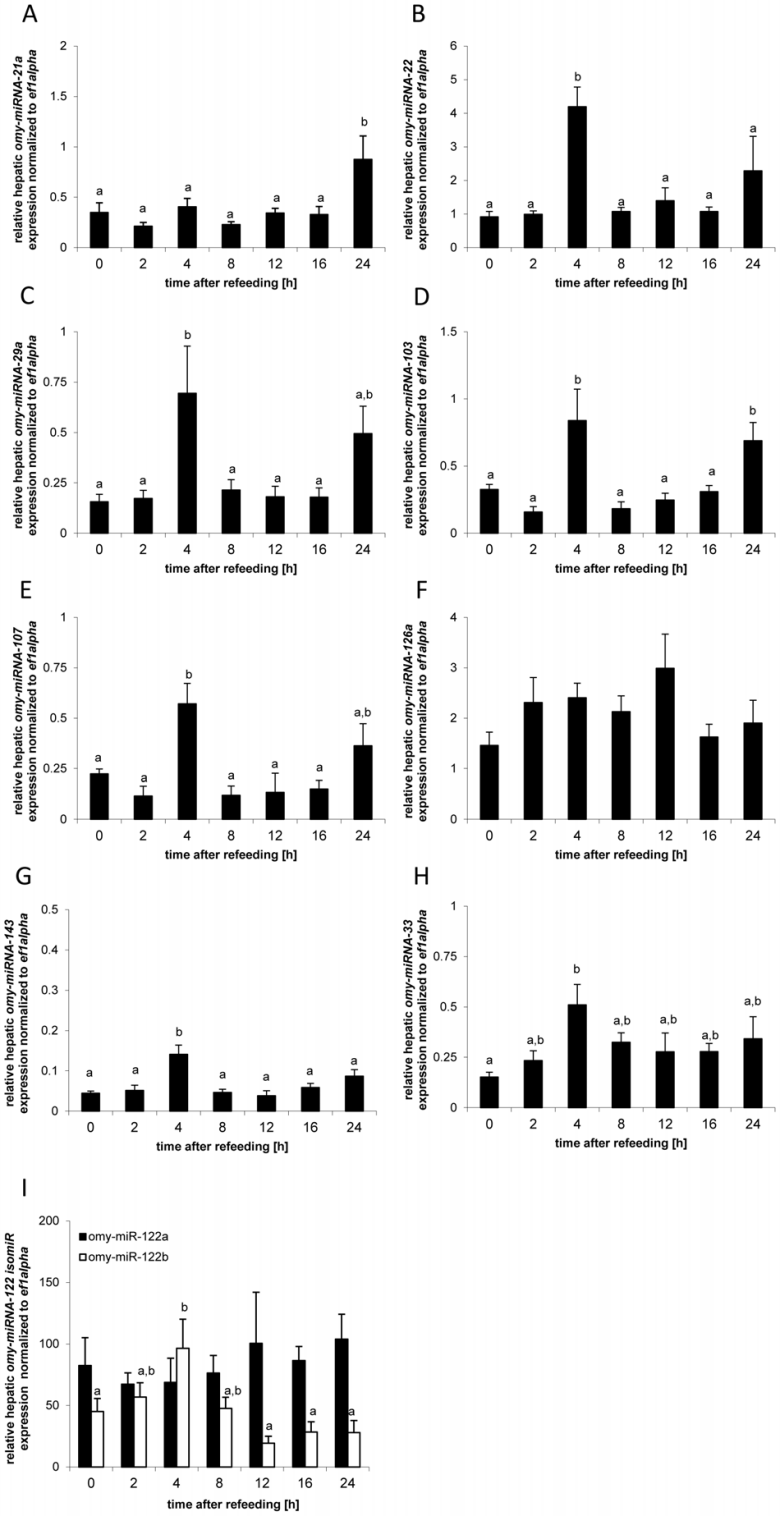
Postprandial expression profiles of hepatic *omy-miRNA* involved predicted to be involved in glucose metabolism (**A**) **and lipid metabolism** (**B**)**.** Means and S.E. of n = 6 samples per group, are shown. Data were analysed using a one-way ANOVA, followed by the Newman-Keuls post-hoc test. Different letters indicate a significant difference at p<0.05.

Time had a significant effect on the expression of *omy-miRNA*s whose mammalian homologues are implicated in the regulation of the insulin signaling cascade, particularly with respect to the regulation of glucose metabolism (**Fig. 2A–G**). The expression of *omy-miRNA-21* (df = 6; F = 4.54; p<0.01), *omy-miRNA-22* (df = 6; F = 5.46; p<0.01), *omy-miRNA-29a* (df = 6; F = 4.35; p<0.01), *omy-miRNA-103* (df = 6; F = 8.06; p<0.01), *omy-miRNA-107* (df = 6; F = 5.59; p<0.01); *omy-miRNA-143* (df = 6; F = 3.79; p<0.05) exhibited significant postprandial changes. A significant increase in expression of several of these miRNAs was observed at 4 h after refeeding when compared to other time points (**Fig. 2B–G**), the exceptions being *omy-miRNA-103* and to some extent *omy-miRNA-29a* and *omy-miRNA-107*, which exhibited an additional second increase in expression after 24 h which was not significantly different from the elevated expression observed 4 h after the meal (**Fig. 2D–E**). No significant postprandial changes in gene expression were observed for *omy-miRNA-126a* (df = 6; F = 1.51; p>0.05). Of the miRNAs, whose mammalian homologues are primarily involved in lipid metabolism, the expression of *omy-miRNA-33* (df = 6; F = 3.41; p<0.05) revealed an increased expression 4 h after the meal when compared to the time of the meal (**Fig. 2H**). Hepatic expression of the highly expressed *omy-miRNA-122b* (df = 6; F = 3.05; p<0.05), but not of *omy-miRNA-122a* (df = 6; F = 0.41; p>0.05), exhibited significant postprandial changes. A significantly higher expression of *omy-miRNA-122b* compared to the time of feeding was observed 4 h after refeeding (**Fig. 2I**).

### Post-prandial Phosphorylation Status of Key Components of the Hepatic Insulin Signaling Pathway

**Figure 3:**
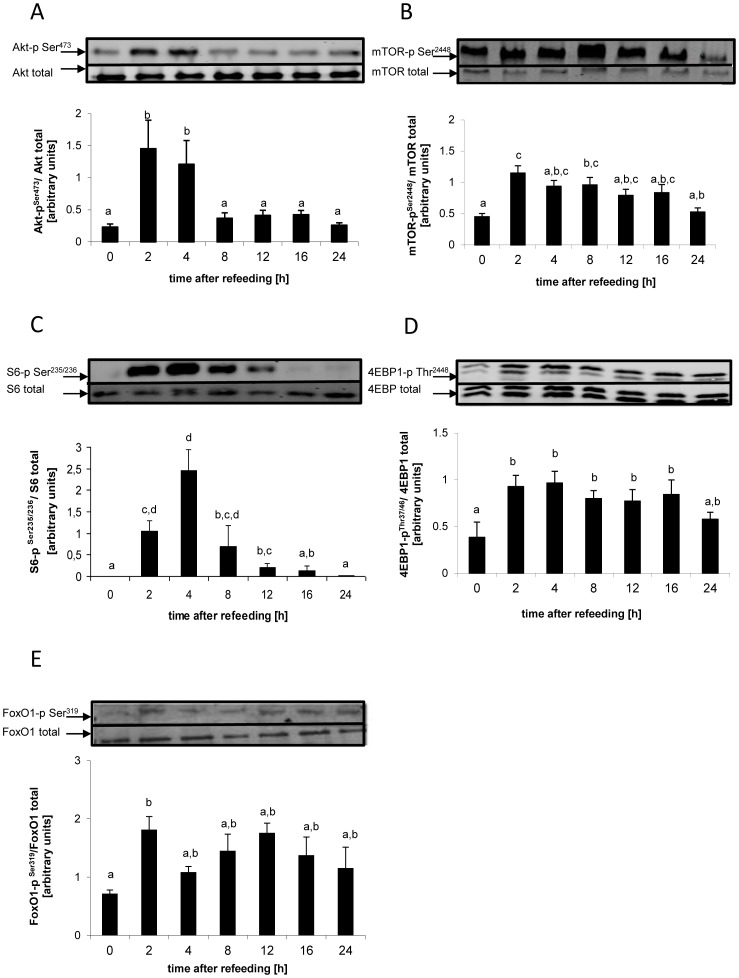
Temporal profile of components of the insulin signaling pathway in rainbow trout, as determined by Western Blot densitometry. Means and S.E. of n = 6 samples per group are shown. Data were analysed using a one-way ANOVA, followed by the Newman-Keuls post-hoc test. Different letters indicate a significant difference at p<0.05.

The postprandial phosphorylation status changed for several components of the insulin signaling pathway in the liver (**Fig. 3A-E**). For the ratio of Akt-p/Akt (df = 6; F = 5.05; p<0.01), a significant elevation in the ratio occurred at 2 h and the ratio returns to basal levels after 8 h (**Fig. 3A**). The phosphorylation ratio for the Akt target mTOR (df = 6; F = 4.18; p<0.01) increased significantly 2 h and 8 h after refeeding (**Fig. 3B**). The mTOR targets S6 (through activation of S6K) and 4-EBP1 equally exhibited postprandial changes in phosphorylation status: for the ratio of S6-p/S6 (df = 6; F = 11.60; p<0.01), a significant postprandial increase was observed between 2 h-12 h, with a maximum 4 h after refeeding (**Fig. 3C**). A significantly elevated ratio of 4EBP1-p/4EBP1 (df = 6; F = 3.6; p<0.01) was equally observed between 2 h and 16 h after refeeding (**Fig. 3D**). The phosphorylation status of FoxO1 (df = 6; F = 3.27; p<0.05) increased significantly 2 h after refeeding compared to the status in fasted animals. (**Fig. 3E**).

### Postprandial Regulation of Genes Involved Intermediary Hepatic Metabolism

**Figure 4:**
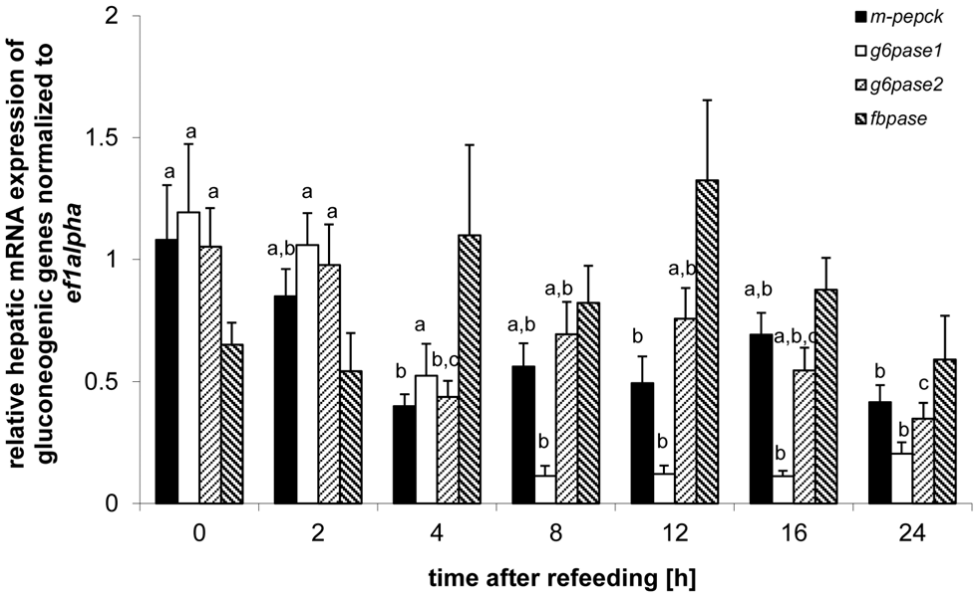
Postprandial regulation of gene expression of hepatic enzymes involved in gluconeogenesis. Means and S.E. of n = 6 samples per group, are shown. Data were analyzed using a one-way ANOVA, followed by the Newman-Keuls post-hoc test. Different letters indicate a significant difference at p<0.05.

Hepatic expression of genes coding for the gluconeogenic enzymes (**Fig. 4**), including *m-pepck* (df = 6; F = 3.96; p<0.01), *g6pase1* (df = 6; F = 16.5; p<0.01) and *g6pase2* (df = 6; F = 5.02; p<0.01) revealed significant postprandial decreases in expression after refeeding, while *fbpase* did not (df = 6; F = 1.68; p = 0.15). The expression of *m-pepck* was significantly decreased 4 h, 12 h and 24 h after refeeding compared to its expression level at feeding time (**Fig. 4**). The expression of *g6pase1* was significantly decreased between 8 h-24 h compared to the expression measured up to 4 h after refeeding (**Fig. 4**). The expression of *g6pase2* was significantly decreased 4 h and 24 h after refeeding compared to its expression at feeding time (**Fig. 4**).

**Figure 5:**
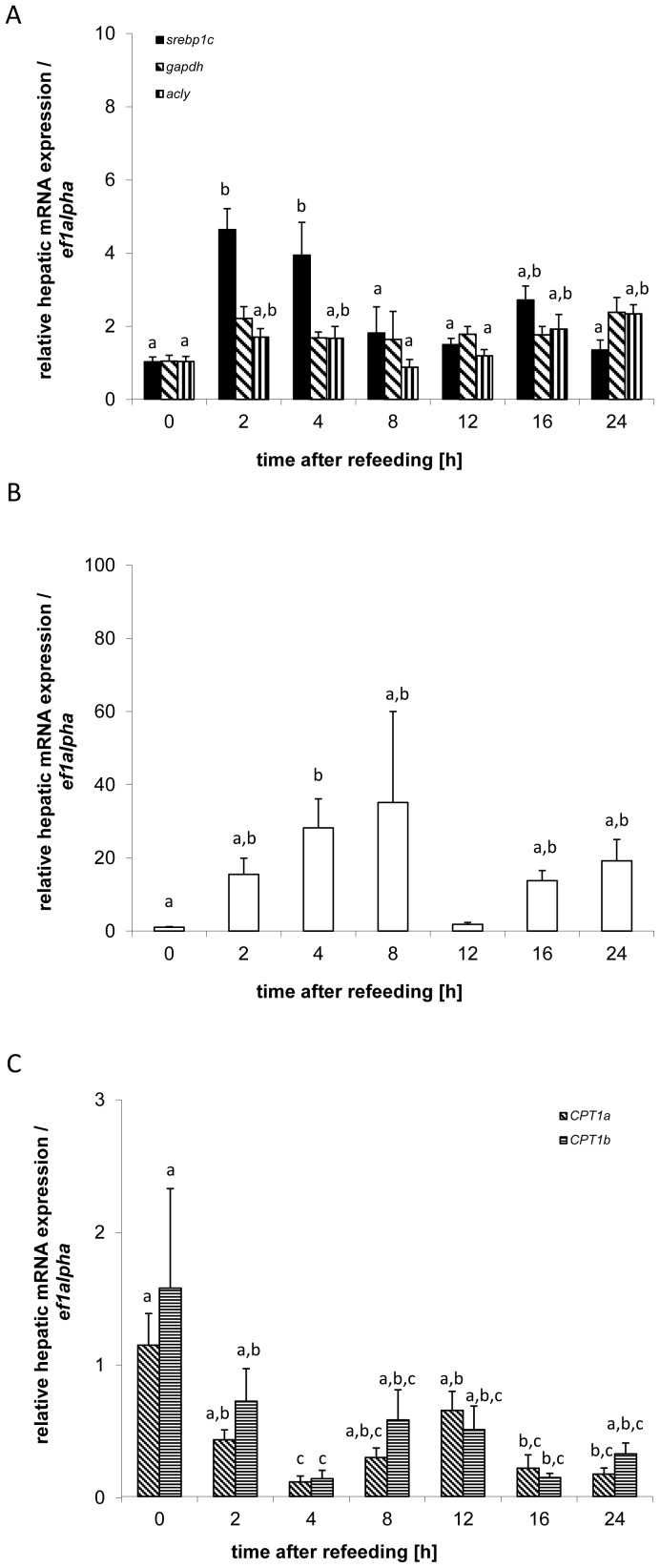
Postprandial expression of genes involved in lipogenesis (A,B) and lipolysis (C) Means and S.E. of n = 6 samples per group, are shown. Data were analysed using a one-way ANOVA, followed by the Newman-Keuls post-hoc test. Different letters indicate a significant difference at p<0.05.

The expression of genes involved in hepatic lipid metabolism increased postprandially for lipogenic genes (**Fig. 5A,B**), including *srebp1c* (df = 6; F = 7.13; p<0.01), *fas* (df = 6; F = 4.41; p<0.01), and *acly* (df = 6; F = 3.74; p<0.01), but not *g6pdh* (df = 6; F = 1.13; p = 0.37). Post-hoc analysis reveals significant increases in the expression of *srebp1c* between 2–4 h after refeeding compared to expression at feeding time, with a second, smaller increase in expression after 16 h that is not significantly different from the expression observed between 2 h and 4 h (**Fig. 5A**). Expression of *fas* increases significantly after 4 h and exhibits a second peak in expression between 16–24 h that is not significantly different from the expression observed 4 h after refeeding (**Fig. 5B**). The postprandial expression of *acly* is significantly increased 24 h after refeeding; however, this increase is preceded by a smaller, first peak in expression between 2–4 h that is not significantly different from the expression observed 24 h after refeeding. The expression of the lipolytic genes (**Fig. 5C**), *cpt1a* (df = 6; F = 5.30; p<0.01) and *cpt1b* (df = 6; F = 4.11; p<0.01), exhibited postprandial decreases in expression. Both isoforms, *cpt1a*, and *cpt1b,* were significantly decreased 4 h and 16 h after refeeding compared to the expression level at the time of refeeding (**Fig. 5C**).

**Figure 6:**
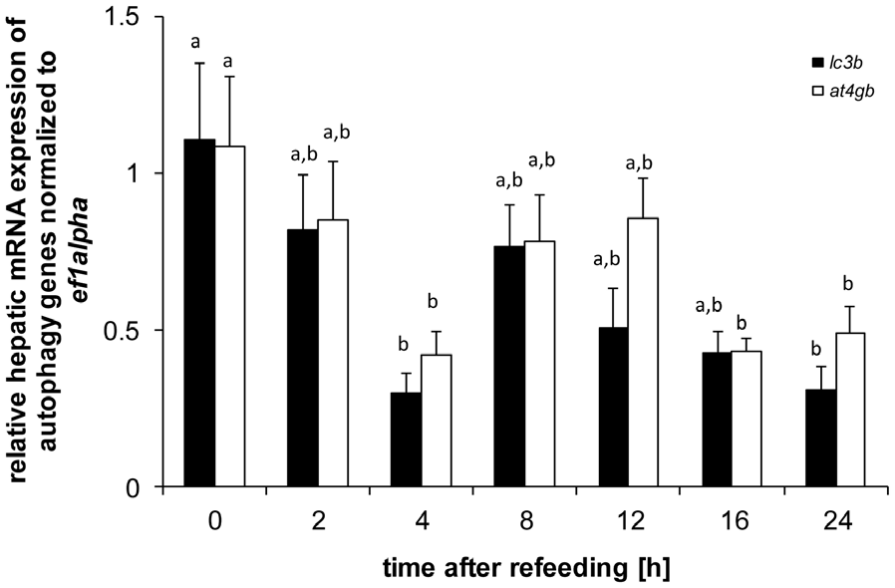
Postprandial hepatic expression of the autophagy genes *at4b* and *lc3*. Means and S.E. of n = 6 samples per group, are shown. Data were analysed using a one-way ANOVA, followed by the Newman-Keuls post-hoc test. Different letters indicate a significant difference at p<0.05.

The hepatic expression of genes with a role in autophagy changed postprandially (**Fig. 6** **)**, as evidenced in the change in gene expression of *atg4b* (df = 6; F = 3.85; p<0.01) and *lc3b* (df = 6; F = 3.38; p<0.01). The expression of both genes significantly decreased at 4 h and 24 h after refeeding (**Fig. 6**).

## Discussion

The results obtained from our postprandial time course study of rainbow trout refed after a short-term fast reveal acute postprandial expression changes of microRNAs in the liver, which are temporarily coordinated with changes in components of the hepatic insulin signaling pathway and mRNA expression of metabolic genes. To our knowledge, this study is the first study to describe the postprandial regulation of hepatic expression of miRNA in any species. The following discussion aims to explore the potential for a role of miRNAs as a new layer of control in the postprandial regulation of the hepatic insulin pathway in rainbow trout.

### Potential Roles for Hepatic Omy-miRNAs in the Negative Feedback of Hepatic Insulin Signaling

Postprandial hepatic metabolism is regulated by insulin in both mammals and fish [33]. The increased phosphorylation of Akt points to insulin activation of its liver signaling pathways between 2–4 h after feeding. The postprandial activation of the hepatic insulin pathway is consistent with persistently elevated plasma insulin concentrations in trout fed the same diet as in the current experiment [41]. The increased phosphorylation of Akt measured in the current study returns to basal levels between 4–8 h therefore implicates the presence of negative feedback mechanism(s) to terminate the initial signaling at the Akt node. Based on mammalian miRNAs known to inhibit the phosphorylation of hepatic Akt (summarized in **Fig. 1**), we investigated the postprandial expression of the homologous omy-miRNAs. Indeed, an increase in postprandial expression of *omy-miRNA-22*, *omy-miRNA-29a*, *omy-miRNA-33*, *omy-miRNA-103*, *omy-miRNA-107* and *omy-miRNA-143* was found 4 h after refeeding, preceding the observed attenuation of Akt phosphorylation status between 4–8 h after the meal. This temporal pattern is in line with the predicted inhibitory effects of these miRNAs on different components of the insulin pathway upstream of Akt, such as IRS and PI3K in both fish and mammals (**Table 1**). These results suggest also that omy-miRNAs may acutely inhibit Akt phosphorylation, similarly to their mammalian counterparts. For example, transient overexpression of *hsa*-*miRNA-29, mmu-miRNA-143*, *mmu-miRNA-103* and *mmu-miRNA-107*, inhibited insulin stimulated Akt phosphorylation in mammalian models *in vitro* and *in vivo* [20], [21], [22]. We equally investigated the expression of *omy-miRNA-21a* and *omy-miRNA-22* based on the fact that their mammalian homologues have a stimulating effect on hepatic Akt phosphorylation via repression of PTEN [23], [24]. Unexpectedly, the expression of *omy-miRNA-22* increased 4 h after the meal, which would result in a stimulation of Akt phosphorylation. This result may indicate that postprandial regulation of miRNAs represents an intricate balance between stimulatory and inhibitory effects on the insulin pathway. An alternative hypothesis is that in rainbow trout, *omy-miRNA-22*, albeit conserved in its sequence compared to mammals, may not directly act on the same gene target, whose sequence conservation is equally important in determining adequate miRNA binding, a fact that has so far been neglected in studies investigating miRNA expression in fish species [63]. The latter hypothesis is supported by the prediction that that miRNA-22 targets PI3K subunits in some fish species (**Table 1**), which would result in an inhibition of Akt signaling in line with the observed decrease in Akt activation after 8 h. Nevertheless, the temporally precise increase in several miRNAs functionally associated with the mammalian insulin signaling pathway points to a potential role for these miRNAs in the acute postprandial regulation of the activity of the insulin pathway in rainbow trout.

### Transient Postprandial Increases of *Omy-miRNA-29a*, *Omy-miRNA-103*, *Omy-miRNA-107* and *Omy-miRNA 143* are Consistent with a Stimulatory Role on the Gluconeogenic Pathway

While the increase in hepatic *omy-miRNA* preceeding the return of Akt phosphorylation status to baseline values are largely in line with the known negative regulation of the hepatic insulin pathway by homologous mammalian miRNAs, the functional consequences of this inhibition on hepatic carbohydrate metabolism appear to differ between trout and mammalian models. Similar to mammalian models [23], [24], activation of the hepatic insulin signaling pathway in trout is correlated with decreased gluconeogenic gene expression for *g6pase1*, *g6pase2*, and *m-pepck*, but not *fbpase2* in our study, confirming previous results showing a rapid inhibition of the transcription and activity of gluconeogenic genes by bovine insulin in trout, again with the exception of FBPase activity [37]. Furthermore, insulin induced activation of Akt and concurrently decreased gluconeogenic gene expression of *g6pase1* and *m-pepck* correlate with increased FoxO1 phosphorylation in trout primary hepatocytes, arguing for an evolutionary conserved mechanism [37].

Assuming a conservation of the action of the homologous miRNAs in trout, the elevated post-prandial expression of *omy-miRNA 29a*, *omy-miRNA-103, omy-miRNA-107* and *miRNA-143* should therefore precede not only an inhibition of hepatic insulin signaling in the form of Akt and FoxO1 phosphorylation, but also preceed a subsequent de-repression of hepatic gluconeogenic gene expression. Indeed, the increase in *omy-miRNA29a, omy-miRNA-103/107* and *omy-miRNA-143* expression 4 h after the meal precedes not only a decrease in Akt and FoxO1 phosphorylation 8 h after the meal, but also a mild, yet significant, attenuation of the initial inhibition of *m-pepck* and *g6pase2* expression. This is evidenced by the fact that the postprandial hepatic expression of the gluconeogenic genes *m-pepck* and *g6pase2* is significantly decreased 4 h following the meal, but not at 8 h following the meal when compared to expression levels in fasted fish (T0). Nevertheless, both the increase in the specific miRNAs at 4 h and the attenuation of the initial repression of gluconeogenic genes at 8 h are transient, as the expression of both *m-pepck, g6pase2* are again significantly inhibited 24 h after the meal, an observation that may be linked to the fact that proteins, the main component in this diet, are stronger activators of the insulin pathway in trout than carbohydrates [33] and can thus cause a stronger repression of gluconeogenic genes [37]. Interestingly, the second decrease in gluconeogenic gene expression after 24 h was again accompanied by a second, albeit smaller increase in *omy-miRNA-103* and *omy-miR-107*, whose expression 24 h after the meal is not significantly different from the previously observed expression peaks 4 h after the meal. This observation strengthens the hypothesis that *omy-miRNA-103* and omy-*miRNA-107* may be involved in a physiological feedback to limit the repression of gluconeogenic gene expression. In this context it is interesting to note that the host-gene of miRNA-103 and miRNA-107, *pank,* codes for a key enzyme whose expression [43] and activity [44] are essential to maintain gluconeogenesis in fasted rats. Therefore, miRNA-103 and miRNA-107 may have evolved to coordinatively support their host genes’ gluconeogenic function by attenuating insulin repression of gluconeogenic gene expression. It will be worthwhile to further test the hypothesized regulatory role of *omy-miRNA-103* and *omy-miRNA-107* by challenging trout with a hyperglycaemic diet (>20% carbohydrates), as the glucose intolerant phenotype in trout has been linked to a constitutively active gluconeogenic pathway in the liver [45], similar to the situation of human type-II diabetic patients [3]. As aberrant expression of the described mammalian miRNA homologues is observed in type-II diabetes and obesity models [5], [6], [7], this may elucidate not only the metabolic phenotype of rainbow trout, but contribute to the understanding of metabolic pathologies. Overall, the postprandial *omy-miRNA* expression pattern is largely consistent with the described role of their mammalian homologues and may acutely interact with the insulin pathway in trout to regulate gluconeogenic gene expression.

### Postprandial Increases in Hepatic *MiRNA-33* and *-122b* Correspond to the Acute Induction of Lipogenic Pathways in the Liver

The second functional group of miRNAs investigated in trout was chosen based on the role of their mammalian homologues on the hepatic insulin pathway and lipid metabolism.

We specifically investigated the postprandial hepatic expression of two miRNAs, *omy-miRNA-33* and *omy-miRNA-122b*, whose mammalian homologues act on the hepatic insulin pathway to stimulate lipogenesis and inhibit lipolysis [46]. A miRNA-33 homologue has only recently been characterized in fish [47] and appears to be similar to isoform *hsa-miRNA-33a* in humans. In humans, two miRNA-33 isoforms exist in two separate *srebp* host genes, where they augment the host gene’s regulation of lipid metabolism [18]. Conversely, two isoforms of miRNA-122, *omy-miRNA-122a* and *omy-miRNA122b*, exist in rainbow trout [33], contrary to the situation in humans, where a single isoform exists. However in both, rainbow trout and higher vertebrates, miRNA-122 is highly abundant and liver specific [48].

To characterize the postprandial regulation of hepatic lipid metabolism we measured the expression of lipogenic (*srebp1c, fas, acly*) and lipolytic genes (*cpt1a*, *cpt1b*) and observed a synexpression of lipogenic genes and concurrent inhibition of lipolytic genes 4 h and 24 h after refeeding. The temporal synexpression of the lipogenic genes confirms previous studies in several fish species [34], [49], [50] and likely points to an evolutionary conserved role for SREBP1 in transcribing key metabolic genes of lipogenesis [51]. However, the concomitantly observed inhibition of the lipolytic genes at both postprandial time points strongly suggests an inverse regulation of both pathways by upstream factors. Both, *omy-miRNA-33* and *omy-miRNA-122b* may represent such upstream factors, as their mammalian homologues promote lipogenesis while simultaneously inhibiting lipolysis by stimulating lipogenic and inhibiting lipolytic gene expression in the liver. For example, inhibition of *mmu-miRNA-33* expression *in vivo* results in lowered plasma VLDL triglyceride levels, and correlates with decreased hepatic gene expression of *srebp1*, *fas* and *acly*, as well as increased expression of *cpt1a* [19]. An inhibition of *cpt1a* expression and a measurable decrease in fatty acid oxidation had also previously been shown in human cell lines overexpressing *hsa-miRNA-33a* and *hsa-miRNA-33b* [18]. Similarly inhibition of *mmu-miRNA-122 in vivo* results in increased fatty acid oxidation and decreased fatty acid synthesis rates and is further correlated with a decrease in *srebp1*, *fas* and *acly* expression [25], [29], [52]. The lipogenic role of miRNA-122 appears to be conserved in fish, based not only on observations in our current study, but also by the detection of a concurrent expression increase of *dre-miRNA-122*, *srebp1c* and *fas* in a transgenic zebrafish model for hepatic steatosis [53]. In our study, *omy-miRNA-33* and *omy-miRNA-122b* increased 4 h after the meal, consistent with the concomitant stimulation of lipogenic genes and inhibition of lipolytic genes. Furthermore, the observed postprandial expression pattern of *omy-mRNA-122b* decreases sharply between 4 h to 12 h after feeding, a decrease which is paralleled by a decrease in *srebp*, *fas* and to some extent *acly*, and a transitory increase in *cpt1a* and *cpt1b,* providing further evidence for a synexpression group. The lowest abundance of *omy-mRNA-122b* occurs at the time of significantly increased plasma concentration of triglycerides (data not shown), an endproduct of the lipogenic pathway. This correlation points to the possibility of an acute nutritional feedback regulation of the triglycerides on *omy-miRNA-122b*, similar to the observation in studies of a mammalian obesity model characterized by increased triglyceride concentrations, which exhibited decreased miRNA-122 expression [5]. Interestingly, *hsa-miRNA-370* has recently been found to equally coordinate lipogenic and lipolytic gene expression in *in vitro* by increasing *hsa-miRNA-122* and subsequent lipogenic gene expression on the one hand, and by inhibiting *cpt1a* expression on the other [54]. However, no fish isoform of miRNA-370 has been characterized to date, but it is tempting to speculate on a similar role in the postprandial regulation of lipogenic genes, at least in higher organisms.

The observed changes in miRNA gene expression are largely consistent with the observed postprandial induction of the lipogenic pathway and may therefore act to augment the lipogenic role of insulin in trout liver [39], which is equally attributed to increased expression of the lipogenic genes *srebp1c*, *fas*, *acly* and a concurrent inhibition in the expression of the lipolytic gene *cpt1a* [37].

### Potential Involvement of MiRNA in Hepatic Autophagy Gene Regulation

Interestingly, the insulin pathway has equally been shown to inhibit hepatic autophagy gene expression in mammals, acting via phosphorylation of mTOR [55] and FoxO1 [2]. In the context of postprandial regulation of hepatic genes it is important to notice that a pathway that has not only been implicated in regulating the availability of intracellular metabolites [56], but also the regulation of systemic macronutrient concentrations, including carbohydrates and amino acids [15], as well as lipids [16]. In our study, we observed an acute postprandial down-regulation of hepatic expression of *atg4b* and *lc3b*, two genes involved in the complex II formation of the autophagosomes [56], 4 h after the ingestion of the meal. This effect correlated with increased activity of the insulin pathway, as evidenced by a concomitant increase in Akt, FoxO1 and mTOR phosphorylation status. Indeed, the observed down-regulation of hepatic autophagy genes is consistent with a conserved inhibitory effect of insulin on the regulation of hepatic autophagy in mammals [2], but is contrary to the lack of effect of insulin on the regulation of these genes in trout muscle [57]. Similarly to the transient effect observed in the repression of gluconeogenic genes described earlier, the repression of *atg4b* and *lc3b* is rapidly attenuated after 8 h. This attenuation is preceded by an increase in the expression of several *omy*-miRNAs (*omy-miRNA- 29a, omy-miRNA-103*, *omy-miRNA-107, omy-miRNA-143*) 4 h after the ingestion of the meal. All of these aforementioned miRNAs are predicted to inhibit elements of the insulin pathway upstream of Akt. Indeed, the expression increase in omy-miRNAs 4 h after the meal is followed by a potent inhibition of Akt phosphorylation status 8 h after the meal. Therefore, while our study largely failed to identify *omy*-miRNAs that may directly inhibit autophagy related genes, a miRNA-dependent regulation of the insulin signaling pathway by specific miRNAs may indirectly contribute to the attenuation of postprandial repression of hepatic autophagy genes in the rainbow trout. A possible direct regulation may however occur through *omy-miRNA-29a*, whose homologues in stickleback and the green-spotted-puffer fish (**Table 1**) are predicted to target the *atg4b* gene. Overall, this is consistent with the prediction of the insulin pathway, as well as of specific miRNAs different from those investigated in our study, as direct regulators of autophagy [58]. With regard to the potential postprandial regulation of the process of autophagy, it is important to note, however, that changes in hepatic *atg4* and *lc3b* gene expression do not functionally prove autophagy in *sensu strictu*, and future studies describing the postprandial functional activity of hepatic autophagy in trout are warranted.

### Conclusion and Future Directions

To our knowledge, this study is the first to investigate acute postprandial regulation of hepatic miRNAs *in vivo* in any species. The observed expression pattern of *omy-miRNA-29a, omy-miRNA-103/107* and *omy-mRNA-143,* as well as *omy-miRNA-33* and *omy*-*miRNA-122b* is largely consistent with an integrated role in the acutely activated insulin pathway. It is interesting to note that the miRNAs chosen for their modulatory role in the mammal insulin pathway were largely stimulated 4 h after refeeding, implicating a potential regulation by the insulin pathway itself. It is however not clear at this point, whether the increase in miRNA abundance is caused by hormonal factors, distinct nutrients or a combination of both. A regulation of miRNAs has been shown for both, insulin [59], but also for nutritional factors, such as amino acids [60], in muscle. While our current postprandial *in vivo* study cannot adequately resolve this point, *in vitro* studies on trout hepatocytes are warranted, to investigate the role/potential interaction of both factors on miRNA regulation.

Additionally, miRNA antagonism studies similar to studies in mammalian systems are required in trout and in fish species in general, in order to validate the predicted miRNA target depicted in **Table 1**. This is especially important with respect to investigate predicted differences in miRNA targets between (human-fish) or within vertebrate classes (fish), both of which are evident in **Table 1**. While miRNAs are evolutionarily highly conserved across animal phyla [61], the recognized sequence in potential target genes may not be, a point which has not been addressed in studies investigating miRNA expression in fish [63]. The estimation of evolutionary conservation of miRNA function between fish and mammals is further complicated by the fact that in some fish species, such as rainbow trout, multiple miRNA isoforms may exist, as exemplified by *omy-miRNA-122a* and *b,* which are differentially regulated in our study [33].

The rainbow trout lends itself as a model to investigate miRNA regulation of hepatic metabolism, especially due to its glucose-resistant phenotype, characterized by a type-II diabetes phenotype, similar to mammals. With the recent identification of key roles of miRNA for this phenotype in mammalian models [21], [22], and the current time course-study, comparative studies of miRNA function in trout are warranted. The study of miRNA in hepatic metabolism has additional importance for trout aquaculture as it will undoubtedly contribute to a better understanding of the nutritional regulation of intermediary metabolism in this tissue. The fact that efforts to characterize miRNAs of rainbow trout [33], as well as other commercially important fish species, such as Barramundi, *Latis calcifer* [62], silver carp, *Hypophthalmichthys molitrix* [47], the Japanese flounder, *Paralychtis olivacaeus* [63], and the Atlantic halibut, *Hippoglossus hippoglossus*, [64] have recently been made underlines the importance of this emerging research area. Our study represents the first characterization of miRNAs in the context of hepatic metabolism in fish, and will therefore provide a framework for future investigations in aquaculture fish species.
